# Supplementary material for: Anaerobiosis and Mutations Can Reduce Susceptibility of Pseudomonas aeruginosa to Tobramycin Without Reducing the Cellular Concentration of the Antibiotic
Source: Pathogens. 2025 Feb 13;14(2):187. doi: 10.3390/pathogens14020187 (PMC11858066; doi:10.3390/pathogens14020187)
Supplement: Supplementary file 1 [file pathogens-14-00187-s001.zip › Supplementary material.zip!/Supplementary material.pdf]

Supplementary material

Table S1. Bacteria used in this study.

Table S2. Values for individual replicates of MICs

Table S3. Values for individual replicates of concentrations of tobramycin in *P. aeruginosa*.
