# Supplementary material for: Anaerobiosis and Mutations Can Reduce Susceptibility of Pseudomonas aeruginosa to Tobramycin Without Reducing the Cellular Concentration of the Antibiotic
Source: Pathogens. 2025 Feb 13;14(2):187. doi: 10.3390/pathogens14020187 (PMC11858066; doi:10.3390/pathogens14020187)
Supplement: Supplementary file 1 [file pathogens-14-00187-s001.zip › Supplementary Table S1.zip!/Supplementary Table S1.pdf]

Supplementary Table S1. Bacteria used in this study.

Pseudomonas aeruginosa **strain**	**Source**	**Reference**
**Wild-type bacteria**		
PAO1	Reference strain	[1]
001C	Clinical isolate	[2, 3]
015A	Clinical isolate	[2, 4]
036-1	Clinical isolate	[2, 4]
046-6	Clinical isolate	[5]
006-A2	Clinical isolate	[2]
**Experimentally-evolved mutants**		
TanA3	PAO1 derivative	This study
TanB3	PAO1 derivative	This study
TanC3	PAO1 derivative	This study
TanD3	PAO1 derivative	This study
TanE3	PAO1 derivative	This study
TanF3	PAO1 derivative	This study
TanG5	PAO1 derivative	This study
TanH4	PAO1 derivative	This study
TanI5	PAO1 derivative	This study
TanJ3	PAO1 derivative	This study
**Engineered mutants**		
PAO1Δ*mexXY*	PAO1 derivative	[6]
015AΔ*mexXY*	015A derivative	[6]
006-A2Δ*mexXY*	006-A2 derivative	[6]

1. Stover CK, Pham XQ, Erwin AL *et al.* Complete genome sequence of *Pseudomonas aeruginosa* PA01, an opportunistic pathogen. *Nature*. 2000; **406**: 959-64.

2. Martin LW, Robson CL, Watts AM *et al.* Expression of *Pseudomonas aeruginosa* antibiotic resistance genes varies greatly during infections in cystic fibrosis patients. *Antimicrob Agents Chemother*. 2018; **62**: e01789-18.

3. Wardell SJT, Gauthier J, Martin LW *et al.* Genome evolution drives transcriptomic and phenotypic adaptation in *Pseudomonas aeruginosa* during 20 years of infection. *Microb Genom*. 2021; **7**: 000681.

4. Ramsay KA, McTavish SM, Wardell SJT *et al.* The Effects of Sub-inhibitory Antibiotic Concentrations on *Pseudomonas aeruginosa*: Reduced Susceptibility Due to Mutations. *Front Microbiol*. 2021; **12**: 789550.

5. Martin LW, Gray AR, Brockway B *et al. Pseudomonas aeruginosa* is oxygen-deprived during infection in cystic fibrosis lungs, reducing the effectiveness of antibiotics. *FEMS Microbiol Lett*. 2023; **370**: 1-8.

6. Thacharodi A, Lamont IL. Aminoglycoside resistance in *Pseudomonas aeruginosa*: the contribution of the MexXY-OprM efflux pump varies between isolates. *J Med Microbiol*. 2022; **71**.
